# T197. A DRD2 CO-EXPRESSION GENE SET ENRICHED FOR SCHIZOPHRENIA RISK GENES IS CHARACTERIZED BY A COMMON TRANSCRIPTIONAL REGULATION INVOLVING NURR1 TRANSCRIPTION FACTOR

**DOI:** 10.1093/schbul/sby016.473

**Published:** 2018-04-01

**Authors:** Silvia Torretta, Antonio Rampino, Giulio Pergola, Maria Pennuto, Manuela Basso, Pasquale Di Carlo, Rita Masellis, Giuseppe Blasi, Alessandro Bertolino

**Affiliations:** 1 University of Bari “Aldo Moro”; 2 University of Trento, University of Padova; 3 University of Trento

## Abstract

**Background:**

Genome-wide association studies demonstrated that multiple genetic variants are associated with schizophrenia (SCZ). Additional evidence revealed that genes are prone to operate in functional molecular networks that subtend complex clinical phenotypes. This knowledge raises the need to investigate how genes linked to SCZ and their possible co-regulators are inserted into molecular networks with a key impact in disease pathogenesis. Using post-mortem brain mRNA data sets (Pergola et al. 2017), we have previously identified a co-expression network enriched for SCZ risk genes, including DRD2, the gene coding for the D2 dopamine receptor, and predicted cognitive and neuroimaging phenotypes of SCZ, as well as response to antipsychotic treatment. Given the relevance of DRD2 to the pathophysiology of SCZ, in the current study we sought to further our understanding of biological mechanisms underpinning co-expression of the DRD2 network. In detail, we aimed at probing the hypothesis that expression of genes within the DRD2-related co-expression network is modulated by a common transcriptional regulation involving one or more Transcription factors (TFs).

**Methods:**

In order to identify TF binding sites (TFBSs) in the promoter region of the 85 genes belonging to the DRD2 co-expression network, we performed a motif enrichment analysis using Pscan and Genomatix MatInspector tools. Biological validation experiments were performed in primary mouse cortical neurons. By real-time PCR analysis we measured the mRNA transcript levels of a group of genes included in the DRD2 co-expression module in basal conditions and upon viral vector-mediated overexpression (OE) and knockdown (KD) of the predicted TFs. We studied expression of genes linked to either DRD2 in the co-expression gene set, such as Gpld1, Chit1, Btg4 and Osr1, or SCZ risk, such as Gatad2a and Slc28a1. We also analyzed transcript levels of Cnr1, which mediates cannabinoid-induced transmission and is relevant to SCZ. Moreover, we analyzed the transcript levels of D2 long splicing isoform (D2L), which was included in the co-expression network, and D2 short splicing isoform (D2S), to verify whether TF regulation was specific for D2L.

**Results:**

Promoters of the DRD2 co-expression gene set were enriched for two TFBSs, recognized by Nur-Related Factor 1 (NURR1, FDR-adjusted p=0.03) and Estrogen-Related Receptor Alpha (ERR1, FDR-adjusted p=0.02), respectively. Validation experiments in mouse primary cortical neurons established that NURR1, and not ERR1, is a regulator of genes of the DRD2 co-expression module analyzed in this study. In detail, Nurr1 gain of function (OE) decreased Cnr1 transcript levels (p=0.0002), whereas it increased Gpld1 transcript levels (p=0.03). The transcript levels of these genes showed an opposite expression trend upon Nurr1 KD. D2L (p=0.008), but not D2S, Gatad2a (p=0.00001), Slc28a1 (p=0.0005) and Chit1 (p=0.02) showed significant expression profile changes only upon Nurr1 OE and vice versa.

**Discussion:**

NURR1 participates in developmental, differentiation and survival processes of dopaminergic neurons. It is implicated in transcriptional modulation of genes involved in dopaminergic transmission, including DRD2, as well as in behavioral phenotypes related to dopaminergic anomalies and reminiscent of SCZ in animal models. Finally, NURR1 genetic variation has been associated with SCZ. Taken together, our results are consistent with previous literature and with the hypothesis that molecular mechanisms responsible for co-expression in DRD2 network involve transcriptional regulation by NURR1. They also suggest reliability of our DRD2 co-expression network, and add new insights on mechanisms linked to DRD2-related molecular ensembles and to SCZ.

